# Neurofilament light (NfL) as biomarker in serum and CSF in status epilepticus

**DOI:** 10.1007/s00415-022-11547-4

**Published:** 2023-01-09

**Authors:** Nils G. Margraf, Justina Dargvainiene, Emily Theel, Frank Leypoldt, Wolfgang Lieb, Andre Franke, Klaus Berger, Jens Kuhle, Gregor Kuhlenbaeumer

**Affiliations:** 1 Department of Neurology, University Hospital Schleswig-Holstein (UKSH), Christian-Albrechts-University (CAU), Arnold-Heller-Str. 3, 24105 Kiel, Germany; 2 Institute of Clinical Chemistry, University Hospital Schleswig-Holstein (UKSH), Christian-Albrechts-University (CAU), Kiel, Germany; 3 Institute of Epidemiology and Biobank PopGen, University Hospital Schleswig-Holstein (UKSH), Christian-Albrechts-University (CAU), Kiel, Germany; 4 Institute of Clinical Molecular Biology, Christian-Albrechts-University of Kiel and University Hospital Schleswig-Holstein, Kiel, Germany; 5 Institute of Epidemiology and Social Medicine, University of Münster, Münster, Germany; 6 Multiple Sclerosis Centre, Neurology, Departments of Head, Spine and Neuromedicine, Biomedicine and Clinical Research, University Hospital Basel and University of Basel, Basel, Switzerland; 7 Research Center for Clinical Neuroimmunology and Neuroscience (RC2NB), University Hospital and University of Basel, Basel, Switzerland

**Keywords:** Status epilepticus (SE), Neurofilament light (NfL), Biomarker, Cerebrospinal fluid (CSF), Neurodestruction

## Abstract

**Objective:**

We explored the potential of neurofilament light chain (NfL) in serum and cerebrospinal fluid as a biomarker for neurodestruction in status epilepticus.

**Methods:**

In a retrospective analysis, we measured NfL in serum and cerebrospinal fluid samples of patients with status epilepticus using a highly sensitive single-molecule array technique (Simoa). Status epilepticus was diagnosed according to ILAE criteria. Additionally, we employed an alternative classification with more emphasis on the course of status epilepticus. We used data from three large control groups to compare NfL in status epilepticus versus neurologically healthy controls.

**Results:**

We included 28 patients (mean age: 69.4 years, SD: 15 years) with a median status duration of 44 h (IQR: 80 h). Twenty-one patients (75%) suffered from convulsive status epilepticus and seven (25%) from non-convulsive status epilepticus. Six patients died (21%). Cerebrospinal fluid and serum NfL concentrations showed a high correlation (*r* = 0.73, *p* < 0.001, Pearson). The main determinant of NfL concentration was the status duration. NfL concentrations did not differ between convulsive status epilepticus and convulsive status epilepticus classified according to the ILAE or to the alternative classification without and with adjusting for status duration and time between status onset and sampling. We found no association of NfL concentration with death, treatment refractoriness, or prognostic scores.

**Conclusion:**

The results suggest that neurodestruction in status epilepticus measured by NfL is mainly determined by status duration, not status type nor therapy refractoriness. Therefore, our results suggest that regarding neurodestruction convulsive and non-convulsive status epilepticus are both neurological emergencies of comparable urgency.

**Supplementary Information:**

The online version contains supplementary material available at 10.1007/s00415-022-11547-4.

## Introduction

Status epilepticus (SE) is one of the most important and common neurological emergencies. The incidence in adults varies in different geographic regions, studies, and age groups between 1.3 and 73.7/100,000/year adults with subgroups of 80 years and older reaching an incidence over 150/100,000 [18]. Immediate and consistent action is required to terminate the ongoing epileptic activity because mortality and disability in SE are high. Mortality rates range from 16 to 25% in adults and increase with patient age [3]. For refractory status epilepticus (RSE), the reported fatality rates range from 16 to 39% in adults [19]. Novy, Logroscino and Rossetti reported that only 63% of the survivors of SE and 21% of the survivors with RSE recovered completely [25]. Experiments in primates show that prolonged generalized convulsive seizures cause neuronal damage [23]. However, if convulsive features of the generalized seizures are suppressed by muscle relaxation with curare, neuronal damage still occurs but is somewhat milder, suggesting that generalized non-convulsive seizures are also neurotoxic [24]. In animal models of strictly focal SE, bilateral brain pathology and remote neuronal damage are rare [5]. In summary, animal models of epilepsy suggest (I) that severe neuronal damage is found in brain regions with excessive neuronal discharges leading to glutamatergic excitotoxicity, and (II) neuronal damage is aggravated by physiological compromise associated with convulsions [22]. However, several recent studies of the treatment of SE show that in contrast to former studies neurodestruction is comparable between convulsive and non-convulsive SE (NCSE) [15, 27, 29]. Current studies note that the mortality is even higher in NCSE than in convulsive SE [15, 18]. This lead to fundamental discussions about the treatment of convulsive SE and NCSE [29].

Neuron-specific enolase (NSE) is to date the most promising neurodestruction biomarker in SE, but different studies showed equivocal results concerning the correlation with status duration and outcome [7, 8, 36, 37]. However, none of the biomarkers has yet been introduced in clinical practice [9]. The restricted accessibility of cerebrospinal fluid (CSF), especially for multiple follow-up examinations limits the use of CSF biomarkers. A recently developed highly sensitive single-molecule array (Simoa) immunoassay allows reproducible and precise measurements of brain-specific proteins, such as neurofilaments [28]. Neuronal death leads to the release of neurofilaments in CSF and blood. Neurofilament light chain (NfL) has been investigated successfully as a biomarker of neurodestruction in various neurological diseases [42, 43].

Here, we measured NfL in CSF and serum of patients with SE using Simoa technology to explore three hypotheses: First, we asked whether the NfL concentrations in SE are increased in comparison to controls. Second, we investigated if the NfL concentrations differ between patients with convulsive SE and patients with NCSE diagnosed by the ILAE classification and an alternative classification placing more attention on the course of the SE. Third, we explored if NfL predicts prognosis and is associated with established predictive scales (STESS, EMSE-EAC), therapy refractoriness, and death.

## Materials and methods

### Setting and study design

This study was conducted in an epilepsy center certified by the German Society for Epileptology (DGfE) at the Department of Neurology of the University Medical Center Schleswig–Holstein (UKSH) in northern Germany. The UKSH, campus Kiel serves a catchment area of about 500,000 people. The study was approved by the Ethics Committee of the Medical Faculty of the Christian-Albrechts-University, Kiel, Germany (D 480/20). The study was conducted following the World Medical Association Declaration of Helsinki. Anonymized data will be shared by request with any qualified investigator.

### Patients with SE

We identified patients with a diagnosis of SE between the 1st of February 2015 and the 15th of May 2020 through the hospital information system of the UKSH. We included all patients aged above 18 years with SE and severely impaired vigilance with available CSF and serum samples in our biobank. We identified a SE according to the International League Against Epilepsy (ILAE) classification described in the “Report of the ILAE Task Force on Classification of SE” [40] and excluded absence status. In subsequent sections, we refer to “convulsive SE” versus “NCSE” and add “according to ILAE” if necessary. We allowed a purely clinical diagnosis for convulsive SE but NCSE required in addition to severely impaired vigilance one of the following EEG phenomena: spike-and-wave complexes, generalized or lateralized periodic discharges (GPDs/LEDs) with additional characteristics according to Trinka and Leitinger [41]. The patients with NCSE included in this study met the Salzburg criteria for NCSE [16].

In addition, we classified patients according to criteria developed by Leitinger and Trinka which take the evolution of semiology into account [18]. In short, patients with a convulsive semiology throughout the SE are categorized as group A, while patients with convulsive semiology at the beginning and later switching to a non-convulsive semiology or purely non-convulsive semiology are in categories B and C which we summarized as one category. We will refer to these categories “convulsive only” and “NCSE at any point during evolution” and add “alternative classification” if necessary [18]. The SE duration was inferred from the complete information available in the hospital information system including the nursing documentation.

We excluded patients with neurological diseases known to lead to significant increases in NfL concentration, namely: brain tumor [10], ischemic or hemorrhagic strokes, septicemia or posthypoxic encephalopathy within the preceding 12 months, cerebral amyloid angiopathy, head trauma, cerebral autoimmune diseases [14], infectious meningoencephalitis [21], severe peripheral neuropathy [e.g., critical illness polyneuropathy before lumbar puncture (LP)] [4]s, and severe neurodegenerative illness [20, 26, 35], e.g., dementia with Lewy bodies (DLB), corticobasal degeneration (CBD), progressive supranuclear palsy (PSP) and amyotrophic lateral sclerosis (ALS).

### Controls

We obtained control measurements of three well-established population-based cohorts. First, we obtained and measured 71 control samples from the Popgen control cohort, a longitudinal study of health in northern Germany [13]. To expand the age range and enlarge the number of control NfL measurements, we obtained 813 additional control values from the population-based arm (cohort 3) of the BiDirect Study [38] and 295 controls values from the MEMO Study, an elderly community sample within the population-based KORA project [32]. We excluded samples with a neurodegenerative disease but not samples with a history of other age-related comorbidities like cardiovascular diseases to avoid the generation of “supercontrols”. The Popgen samples were measured concurrently with the patient samples on the same instrument while BiDirect and MEMO samples were measured on an identical but not the same instrument using the same chemistry.

### Clinical data

We extracted the following clinical data from the medical records: age, sex, date and reason of hospital admission, pre-existing diseases, medication during the hospital stay, duration of SE, time from status onset to sampling, semiology and course of SE, the patient’s outcome assessed in prognostic scores and findings in EEG, cranial MRT and CCT investigations.

### NfL measurements

We obtained all serum/CSF pairs at the same time point, namely the time of LP. Polypropylene tubes were used for CSF and serum. We used tubes with gel separators for blood collection. The serum was separated by spinning at 2000×*g* for 10 min after coagulation. All samples were frozen at − 80 °C in polypropylene cryovials within maximal 48 h at 4 °C. Samples were thawed only once immediately before the measurements.

Analysis was performed on a fully automated HD-X platform (Quanterix) based on Simoa assay technique using a commercially available multiplex kit (Neurology 4-plex: NfL, Tau, UCHL-1, and GFAP). For each run, 8 calibrators were measured in duplicates and two controls (low and high, included in the kit) were analyzed in siglicates at the beginning and end of the run for continuous process control. All samples were measured in duplicates. The coefficients of variance between the replicates of 10% for both serum and CSF were tolerated, by values exceeding 10% the measurements were repeated. For analysis, mean concentration of two replicates was estimated. The laboratory personnel was blinded to the clinical history and other laboratory parameters of the study participants. We analyzed only the NfL measurements of the 4-plex Quanterix kit because NfL is already a fairly well-established biomarker [42, 43] with a large number of available control cohorts and shows favorable properties concerning short transfer time to CSF and blood, the long half-life, and well-developed precise assay [28, 34]. Two patients had missing CSF NfL values because samples had been used up completely. The Shapiro–Wilk test for normality showed that the variables time between status onset and lumbar puncture (“status onset time to LP”) and NfL concentration in CSF as well as serum required logarithmic transformation to conform with the assumption of a normal distribution.

### Prognostic parameters

To estimate the severity of the SE, we used the following outcome scores: SE Severity Score (STESS), modified STESS (mSTESS), and Epidemiology-Based Mortality Score in SE (EMSE) [17, 30, 31]. The STESS rates the categories consciousness, worst seizure type, age, and history of previous seizures with points: 0–3 points predicting a favorable and 4–6 points predicting an unfavorable outcome. There are different versions of the EMSE: We used the EMSE-EAC rating the patient’s etiology, age, and each comorbidity, which leads to an open upwards point scale with 27 points or more predicting high mortality.

### Statistics

We used R/RStudio (version 1.5.46) for all analyses—commands in this paragraph shown in parentheses—and assessed cross-tables using the Fisher exact test (fisher.test). We used the Shapiro–Wilk (shapiro.test) test to assess consistency with the assumption of a normal distribution and calculated mean (mean) and standard deviation (SD) for normally distributed variables. We compared normally distributed variables between two groups with *t* tests (t.test) or the linear model (lm) command equivalent to a *t* test. For variables not following a normal distribution, we calculated median (median) and interquartile ranges (IQR) and compared them between groups with the Mann–Whitney *U* test (wilcox.test). Some variables were log-transformed to achieve consistency with a normal distribution for statistics requiring normally distributed variables, e.g., linear regression (lm) and correlation analysis (cor) using the Pearson method. We calculated receiver operating characteristics using the package pROC (version 1.18.0) and extracted the areas under the curve (AUC). Boxplots display the median as a horizontal line, the hinges indicate the interquartile range (IQR), and the whiskers extend to the smallest/largest value at most 1.5 * IQR of the hinges.

## Results

### Clinical data of the patients with SE

We included 28 patients (24 women, 4 men) with SE and a mean age of 69.4 years (SD =  ± 15)). The median status duration was 44 h (IQR 80 h) and 22 patients (79%) survived SE. Patient characteristics including basic information regarding NfL levels are listed in Table 1. Twenty-one patients (75%) suffered from convulsive SE and seven (25%) from NCSE according to the ILAE classification. All patients showed impaired consciousness. Five patients had epilepsy before the event [unclassified epilepsy *n* = 2 (7%), structural epilepsy *n* = 3 (11%)]. Twelve of the remaining patients had a potential structural cause of SE (43%) and the etiology remained unknown for 11 patients (39%) and 6 of them fit the criteria of NORSE (new-onset refractory status epilepticus) [11]. Fifteen patients (54%) had normal standard parameters in the CSF investigation. Ten patients showed mild to moderate brain–blood-barrier dysfunction, two showed an inflammatory CSF, and one of them was due to an isolated elevated cell count. In 21 patients, the SE was refractory to treatment with a benzodiazepine and a classic antiepileptic drug (RSE, 75%) [2]. Burst suppression was used in 4 patients, either by propofol alone (*n* = 2) or by a combination of propofol and midazolam (*n* = 2). Six patients (21%) died in the hospital. The reasons for death were respiratory failure, peritonitis, cardiac failure, pneumonia, cardiogenic shock, and aspiration pneumonia.

**Table 1:** Basic characteristics of the SE patients and controls

Characteristics	SE patients	Controls	Significance
Sample size	*n* = 28	*n* = 1180	
Sex, *n* (%)			
Male	4 (14%)	600 (51%)	*p* < 0.001^1^
Female	24 (86%)	580 (49%)	
Age, years			
Mean value (± SD)	69 (± 16)	58 (± 12)	*p* < 0.001^1^
Serum NfL, pg/ml			
Mean value	244	12	*p* < 0.001^1^
Median	99	10	
IQR	169	7	
Status duration			
Median in h	44		
≤ 24 h, *n* (%)	13 (46%)	–	
> 24 h, *n* (%)	15 (54%)	–	
Status onset time to LP			
Median in h	72		
Semiology, *n* (%)			
Convulsive SE (ILAE)	21 (75%)	–	
NCSE (ILAE)	7 (25%)		
Convulsive only (alternative classification)	15 (54%)	–	
NCSE at any point during evolution (alternative classification)	13 (46%)		
Refractoriness, *n* (%)			
Responsive	7 (25%)	–	
Refractory	19 (68%)	–	
Super-refractory	2 (7%)		
Etiology, *n* (%)			
Structural	15 (54%)	–	
Unknown	13 (46%)	–	
STESS (0–6)			
Median	4.5	–	
IQR	2	–	
mSTESS (0–8)			
Median	6	–	
IQR	2	–	
EMSE-EAC (cut-off 27)			
Median	40	–	
IQR	31	–	
Died, *n* (%)	6 (21%)	–	

*EMSE-EAC* epidemiology-based mortality score in status epilepticus using the combination of etiology, age and comorbidity, *LP* lumbar puncture, *mSTESS* modified STESS, *NCSE* non-convulsive status epilepticus, *NfL* neurofilament light, *SE* status epilepticus, *STESS* status epilepticus severity score

### NfL in status epilepticus versus control samples

**Fig. 1:**
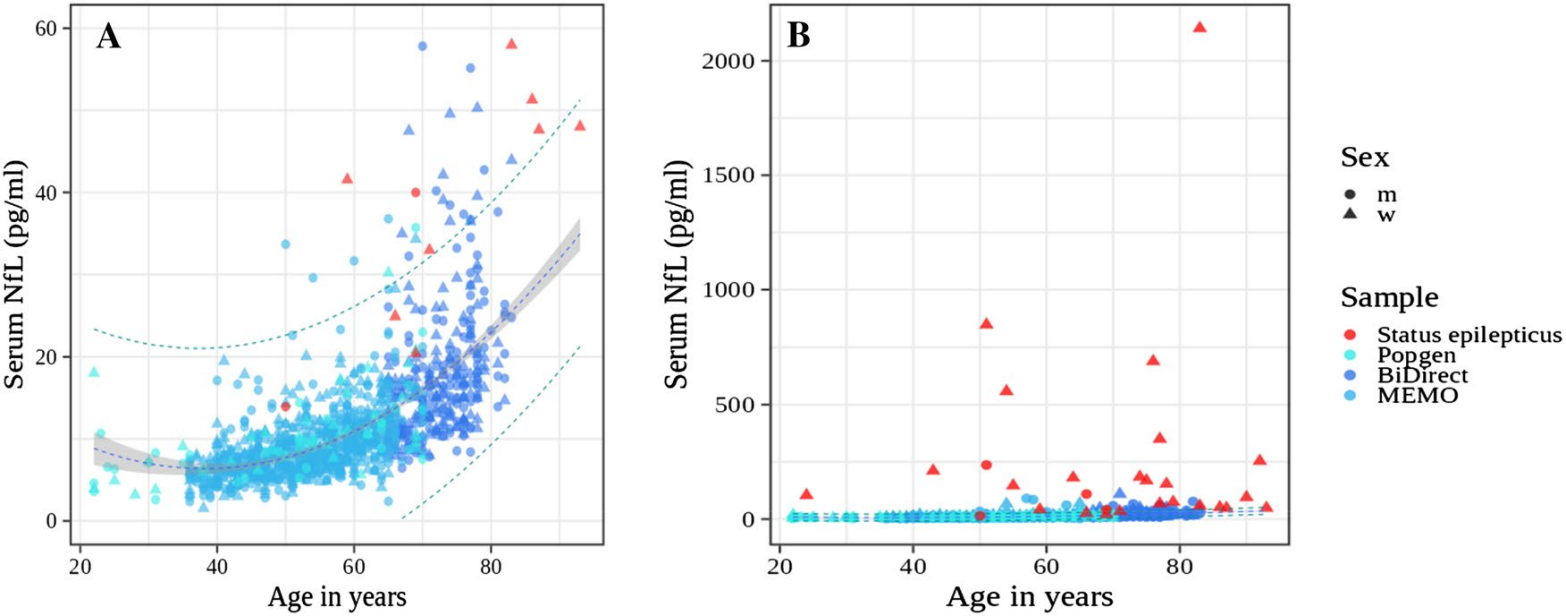
**A** NfL in patients with serum NfL < 60 pg/ml and controls, **B** in all patients and controls. Sample: Status epilepticus = SE patients from this study, Popgen = Popgen control sample measured on the same instrument concomitantly with the epilepsy samples, BiDirect, and MEMO: additional control samples measured on an identical but not the same instrument using the same Simoa kit. Blue interrupted line: NfL per age regression line for the joint control sample, gray band: standard error for the mean of the NfL per age regression line. Green interrupted lines: 95% prediction interval for the NfL per age regression, meaning that the linear model predicts that 95% of all population control samples will show NfL concentrations within these limits

We compared the serum NfL values in SE versus a large set of controls (*n* = 1186, Fig. 1). The control samples covered an age range from 20 to 83 years. Figure 1 shows that the NfL concentration of the Popgen, BiDirect, and MEMO control samples are all in the same range taking age into account. Therefore, we decided to pool all three control samples for analysis. Since NfL did not differ between sexes (*p* = 0.47, *t* test), but increases with age, we fitted a linear model with a quadratic term for serum NfL versus age and determined the 95% prediction intervals and extrapolated to the age of the oldest patient with SE (93 years) (Fig. 1). Only four out of 28 patients with SE showed NfL concentrations within the 95% NfL prediction interval for controls (Fig. 1). In most patients with SE the NfL concentrations grossly exceeded those of controls up to an approximately 100-fold increase for the highest NfL value in a patient (2142 pg/ml).

### Determinants of NfL concentrations in status epilepticus

**Fig. 2:**
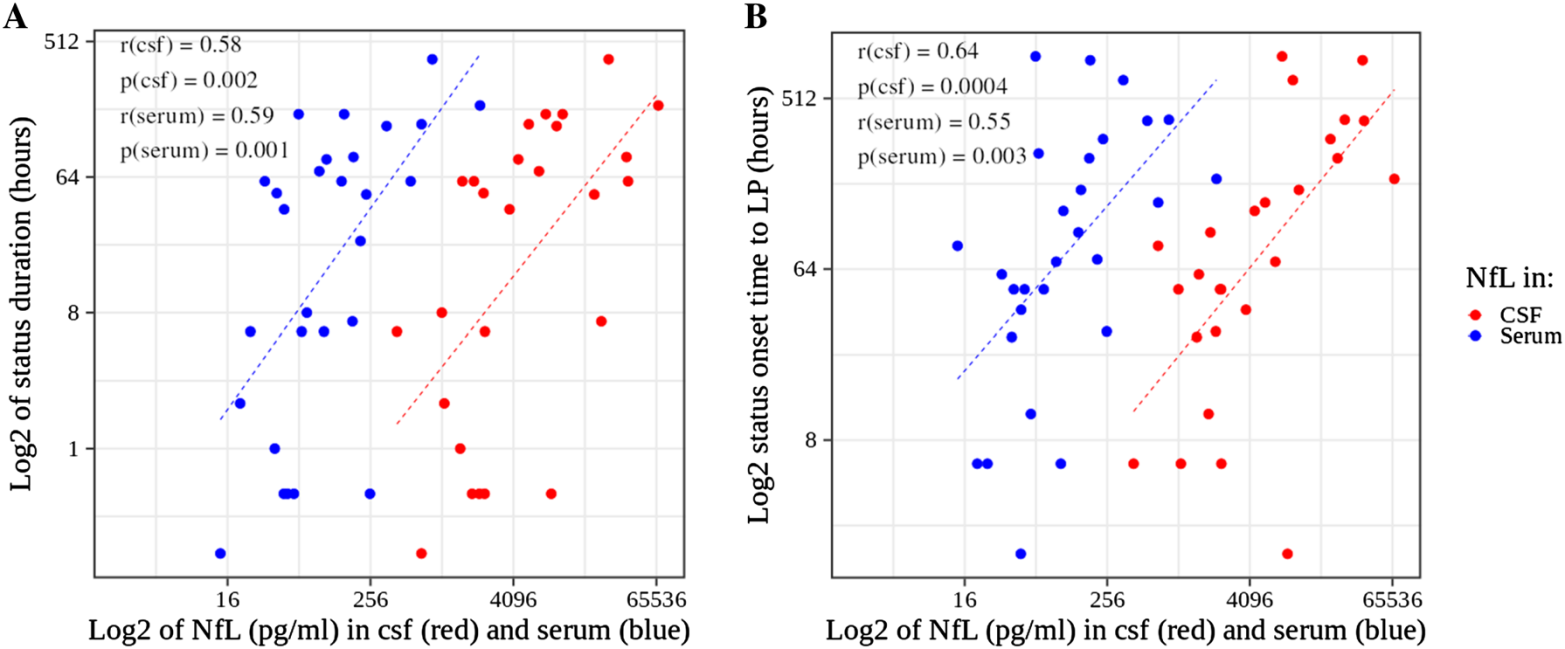
NfL versus **A** status duration and **B** status onset time to LP. *r* Pearson correlation coefficient, *p* *p* value for the correlation

**Fig. 3:**
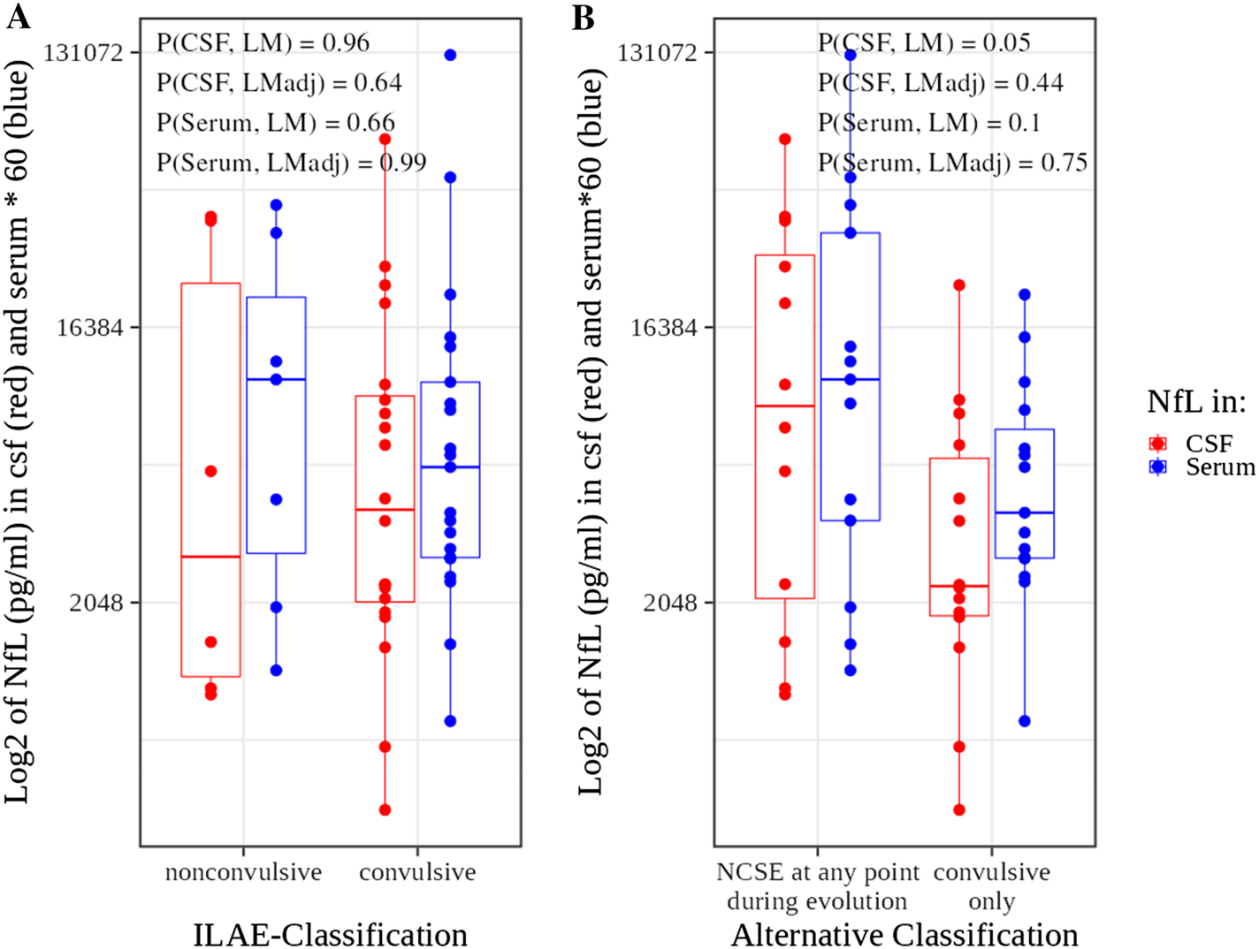
NfL in convulsive SE and NCSE according to: **A** ILAE classification, **B** alternative classification. *p* *p* value, *LM* linear model (equivalent to *t* test), *LMadj* linear model adjusted for status duration and status onset time to LP. Serum NfL concentrations were multiplied by the factor 60 to allow plotting them side by side with the CSF NfL concentrations

Table 2 shows the basic statistic measures presented in Table 1 stratified by the two different classifications of SE differentiating convulsive SE from NCSE and by survival. CSF and serum NfL concentrations showed a high correlation (*r* = 0.73, *p* =  < 0.001, Supplementary Fig. 1). Both, NfL in CSF and serum were correlated with “status duration” (CSF: *r* = 0.58, *p* = 0.002; serum: *r* = 0.59, *p* = 0.001) and “status onset time to LP" (CSF: *r* = 0.64, *p* < 0.0004; serum: *r* = 0.55, *p* = 0.003, Fig. 2). Almost identical slopes of the regression lines for CSF and serum versus "status duration" and "status onset time to LP" indicate an identical relative increment of NfL concentrations in CSF and serum despite differing absolute values, arguing against a large influence of passage time between CSF and serum. "Status duration” and "status onset time to LP" were correlated with each other (*r* = 0.66, *p* = 0.0001, Supplementary Fig. 2), and “status onset time to LP” was always longer or equal to “status duration”. To account for the influence of these covariates (hereinafter referred to only as "covariates") we adjusted for them using linear regression analyses. Without and with adjustment for covariates, NfL concentrations did not differ significantly between patients with convulsive SE and NCSE according to the ILAE classification and the “convulsive only” versus “NCSE at any point during evolution” categories of the alternative classification (Fig. 3). Unadjusted NfL showed a trend towards higher concentrations in treatment RSE (*p*(CSF) = 0.05, *p*(serum) = 0.08). The status duration was longer in treatment RSE than in interruptible SE (mean 86.0 vs. 2.4 h).

**Table 2:** Basic characteristics of the SE patients stratified by epilepsy classification and death

ILAE classification	convulsive SE	NCSE	*p* value (test)
Number	21	7	0.001* (Fisher)
Age (yrs ± SD)	70 ± 18	67 ± 13	0.563 (*t* test)
Sex (number female)	17	7	0.545 (Fisher)
status duration (hrs (IQR))	39 (1–84)	60 (16–74)	0.523 (MWU)
status to LP (hrs (IQR))	85 (30–247)	72 (55–267)	0.750 (MWU)
NFL (csf) (pg/ml (IQR))	4149 (2060–9755)	3518 (1184–28,898)	0.836 (MWU)
NFL (serum) (pg/ml (IQR)	95 (48–180)	184 (54–384)	0.641 (MWU)
Died (Number)	5	1	1.00 (Fisher)
STESS (0–6 (IQR))	5.00 (4.00–6.00)	6.00 (4.50–7.00)	0.634 (MWU)
mSTESS (0–8 (IQR))	7.00 (6.00–8.00)	8.00 (6.50–8.00)	0.382 (MWU)
EMSE-EAC (0–64 (IQR))	35.00 (25.00–47.00)	40.00 (34.50–62.50)	0.457 (MWU)

Alternative classification	convulsive only	NCSE at any point during evolution	*p* value
Number	15	13	0.790 (Fisher)
Age (yrs ± SD)	71 ± 19	67 ± 13	0.463 (*t* test)
Sex (number female)	12	12	0.600 (Fisher)
status duration (hrs (IQR))	6 (1–55)	60 (24–168)	0.011* (MWU)
status to LP (hrs (IQR))	50 (29–115)	168 (60–390)	0.088 (MWU)
NFL (csf) (pg/ml (IQR))	2319 (1853–6167)	9148 (2146–28,639)	0.085 (MWU)
NFL (serum) (pg/ml (IQR)	67 (48–128)	184 (63–558)	0.155 (MWU)
Died (Number)	3	3	1.00 (Fisher)
STESS (0–6 (IQR))	6.00 (4.50 – 6.00)	5.00 (4.00–6.00)	0.865 (MWU)
mSTESS (0–8 (IQR))	7.00 (6.00–8.00)	7.00 (6.00–8.00)	0.635 (MWU)
EMSE-EAC (0–64 (IQR))	43.00 (32.00–51.00)	35.00 (25.00–57.00)	0.519 (MWU)

Survival	Died	Survived	*p* value
Number	6	22	< 0.001* (Fisher)
Age (yrs ± SD)	75 ± 11	68 ± 17	0.267 (*t* test)
Sex (number female)	4	20	0.192 (Fisher)
status duration (hrs (IQR))	50 (14–78)	37 (3–83)	0.674 (MWU)
status to LP (hrs (IQR))	50 (41–113)	93 (50–300)	0.251 (MWU)
NFL (csf) (pg/ml (IQR))	3077 (1729–4324)	6120 (1887–12,883)	0.790 (MWU)
NFL (serum) (pg/ml (IQR)	44 (35–94)	125 (59–230)	0.214 (MWU)
Died (Number)	na	na	na
STESS (0–6 (IQR))	6.00 (6.00–6.00)	5.00 (4.00–6.00)	0.117 (MWU)
mSTESS (0–8 (IQR))	7.00 (7.00–7.75)	7.00 (6.00–8.00)	0.372 (MWU
EMSE-EAC (0–64 (IQR))	61.00 (39.75–81.50)	37.50 (26.00–46.50)	0.123 (MWU)

*EMSE-EAC* epidemiology-based mortality score in status epilepticus using the combination of etiology, age and comorbidity, *LP* lumbar puncture, *mSTESS* modified STESS, *na* not available, *NCSE* non-convulsive status epilepticus, *NfL* neurofilament light, *SE* status epilepticus, *STESS* status epilepticus severity score

### Association of NfL with death and prognostic scales

**Fig. 4:**
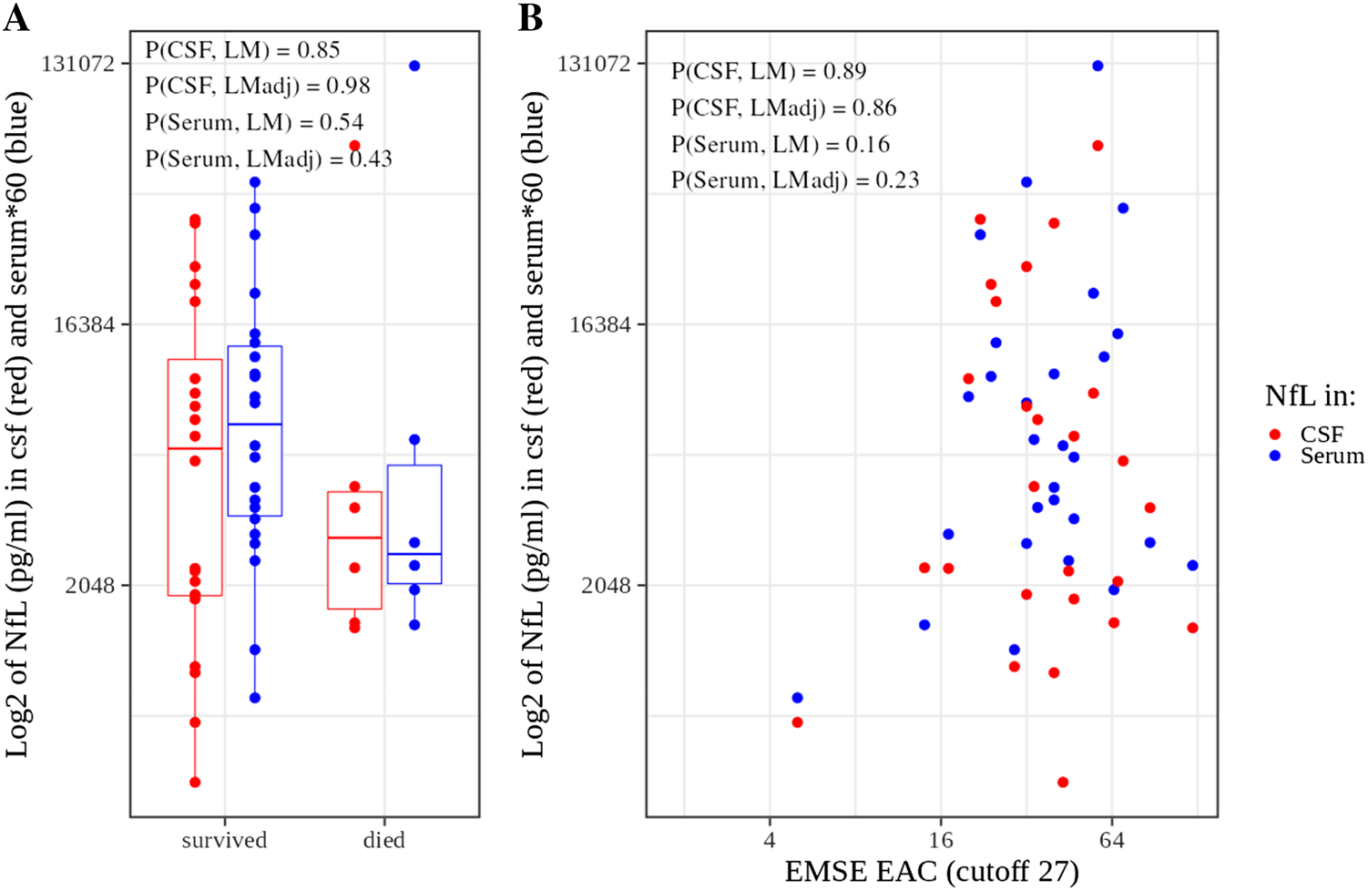
NfL in **A** patients who survived or died, **B** versus EMSE-EAC score. *p* *p* value, *LM* linear model (equivalent to *t* test), *LMadj* linear model adjusted for status duration and status onset time to LP. Serum NfL concentrations were multiplied by the factor 60 to allow plotting them side by side with the CSF NfL concentrations

Next, we assessed the correlation between NfL, death (Fig. 4a) and the prognostic scales EMSE_EAC (Fig. 4b) and STESS/mSTESS (Supplementary Fig. 3). NfL concentration were not associated with death without adjustment for the covariates (*p*(CSF) = 0.78, *p*(Serum) = 0.21) nor with adjustment (*p*(CSF) = 0.98, *p*(Serum) = 0.43, Fig. 4a). Next, we estimated the relation between the STESS and EMSE_EAC and death versus survival using Receiver-Operating-Characteristics (ROC) analysis. We obtained Areas-Under-the-Curve of 0.70 for STESS, 0.62 for mSTESS and 0.71 for EMSE-EAC (data not shown). These values point in the expected direction and are roughly comparable to other studies [12, 33]. We assessed the log-transformed EMSE as an interval-scaled normally distributed variable and STESS (7 levels) as well as mSTESS (9 levels) as ordinal categorical variables. EMSE scores were not correlated with NfL (Fig. 4b) neither without covariate adjustment (*p*(CSF) = 0.89, *p*(Serum) = 0.16) nor with adjustment (*p*(CSF) = 0.86, *p*(Serum) = 0.23).

Due to the low number of individuals in some categories, a meaningful statistical analysis of the STESS and mSTESS data was not possible. However, Supplementary Fig. 3 shows that a trend towards higher NfL values in higher STESS or mSTESS categories is not apparent.

## Discussion

Unlike most discussions, we start with the evident weaknesses of our study, because these are important not only for the interpretation of the data and results but also for the design of follow-up studies. Apart from the cross-sectional design and the relatively small patient sample, the major weaknesses are the large differences in the time to blood/CSF sampling and the high phenotypic variability of SE concerning the etiology, semiology, and duration. While the time to sampling can be standardized, the large phenotypic variability cannot. If standardization of time to sampling leads to NfL as a clinically useful prognostic marker remains to be shown. However, using a standardized sampling time, some status will be sampled after the end of the status, some during the status, and some status will continue a long time after sampling, continuing to release markers of neurodestruction. Despite its weak points, our study allows several meaningful conclusions. First, we show that Simoa technology is sensitive and reliable enough to measure NfL in serum as a proxy for CSF in SE. This is a finding in line with the other paper on that topic by Giovannini and colleagues [6]. In both studies, even though Giovannini used a different method to measure NfL, the correlation between CSF and serum NfL values in SE patients is very similar.

Second, we conclude from the parallel increase in concentration in serum and CSF that the passage time from CSF to blood must be too short to be meaningful even if the time from seizure to sample ascertainment is as short as ~ 2–6 h. We observe a log/log-linear increase of NfL in CSF and serum with status duration as well as time to sampling. We cannot tease apart with certainty whether status duration or time to sampling is the decisive parameter but we think that status duration has higher importance, at least if the sample was taken later than ~ 24 h (15 samples) because studies of “single hit” events like traumatic brain injury have shown that the NfL levels are rather stable after one day [1]. This observation from our study is relevant for any further use of NfL in clinical settings. Early transfer of CSF into serum means that measurement in serum is meaningful. In addition, the measurement of NfL in serum is easy to perform and repeat. Medically, our most important conclusion is that brain damage as measured by NfL might not differ by a very large extent between convulsive SE and non-convulsive SE, at least in patients with severely impaired consciousness. In our study, this is true for SE classified according to the ILAE classification but also for the alternative classification taking the development of the semiology over time into account. This finding suggests that concerning brain damage only, both convulsive, as well as non-convulsive SE, might turn out to be medical emergencies of comparable urgency if our results are corroborated by future studies. This conclusion is in agreement with animal studies as outlined in the introduction but has not yet been shown in humans [5, 22–24]. It is currently a matter of debate if both convulsive- and non-convulsive SE should be treated aggressively considering the adverse effects of sedatives and anticonvulsants [44]. Especially in the elderly, non-convulsive SE is commonly treated less aggressively than convulsive SE [29, 39]. If future studies confirm that non-convulsive SE too leads to significant neurodestruction, intensified treatment might be indicated. Additional findings are that NfL concentrations in most SE patients are higher than the age-adjusted 95th percentile of neurologically healthy controls which is in agreement with the only other study of NfL in SE [6]. Interestingly, the range of NfL concentrations overlapped between SE and controls also in this study [6]. The comparison of NfL between interruptible and treatment RSE pointed towards a possible difference in NfL concentrations just failing to reach statistical significance. However, we think that the main determinant of NfL concentrations is the status duration and not treatment refractoriness because (1) status duration was—as expected—much longer in treatment RSE and (2) status duration was highly linearly correlated on a log–log scale with NfL concentration across the whole range of status durations (Fig. 2). We found no association of NfL levels with scales predicting mortality nor with treatment refractoriness. We cannot exclude that this is caused either by the extreme heterogeneity of SE or the small sample size or our clinical sampling scheme hindering the detection of small differences. This important difference to the study by Giovannini and colleagues might in part be explainable by the fact that we adjusted for the status duration and time to CSF/serum sampling in all analyses while Giovannini and colleagues did not [6]. Larger studies taking the lessons from this study and the study by Giovannini et al. in the study design into account are required to draw firm conclusions. The integration of patients with differential diagnoses of SE is required to explore the diagnostic value of NfL in SE.

## Conclusion

In summary, we show that NfL might be a clinically useful biomarker of SE, that neurodestruction, as measured by NfL, does not differ largely between convulsive SE and NCSE, and point out several caveats and suggestions for subsequent studies.

## Supplementary Information

Below is the link to the electronic supplementary material.

Supplementary file1 (DOCX 491 kb)

## Data Availability

Anonymized data will be shared by request from any qualified investigator.
