# Supplementary material for: Neurofilament light (NfL) as biomarker in serum and CSF in status epilepticus
Source: J Neurol. 2023 Jan 9;270(4):2128–38. doi: 10.1007/s00415-022-11547-4 (PMC10025237; doi:10.1007/s00415-022-11547-4)

**Supplementary Figure 1:** Correlation between NfL in serum and csf


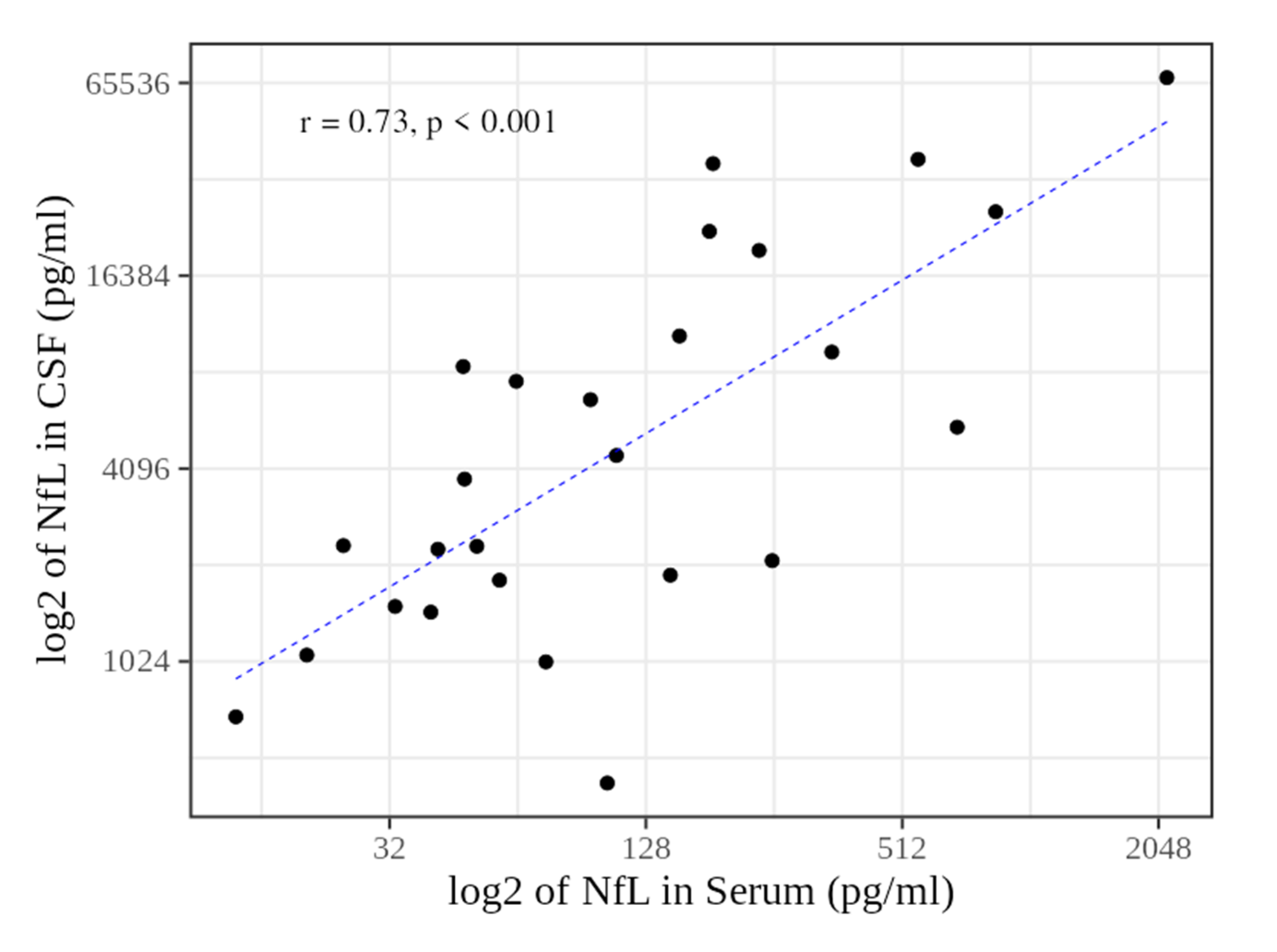


Supplementary Figure 1: r = Pearson correlation coefficient, p = p-value for the correlation

**Supplementary Figure 2:** Correlation between status duration and status onset time to LP


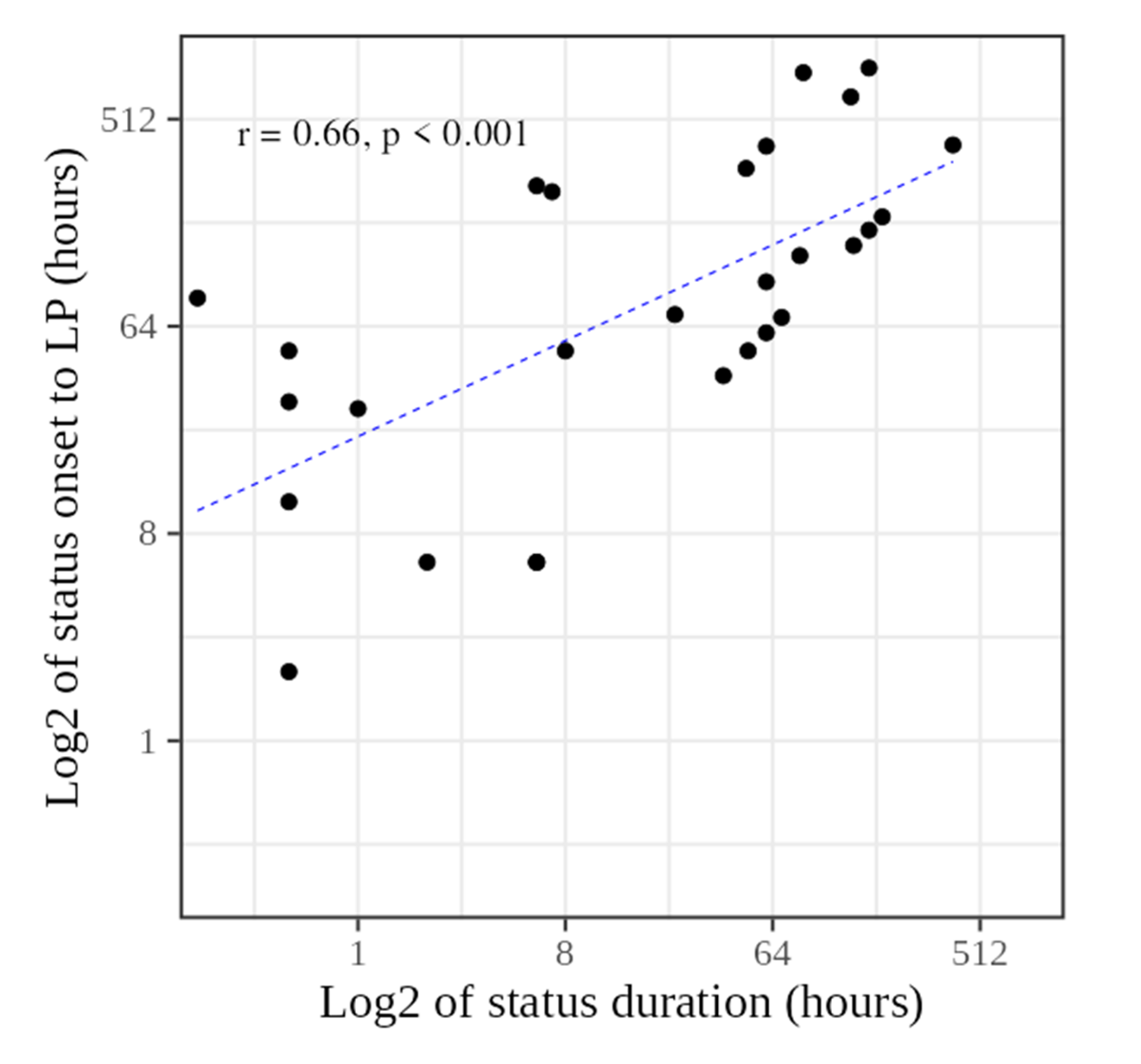


Supplementary Figure 2: r = Pearson correlation coefficient, p = p-value for the correlation

**Supplementary Figure 3:** NfL concentration versus (A) STESS, (B) mSTESS


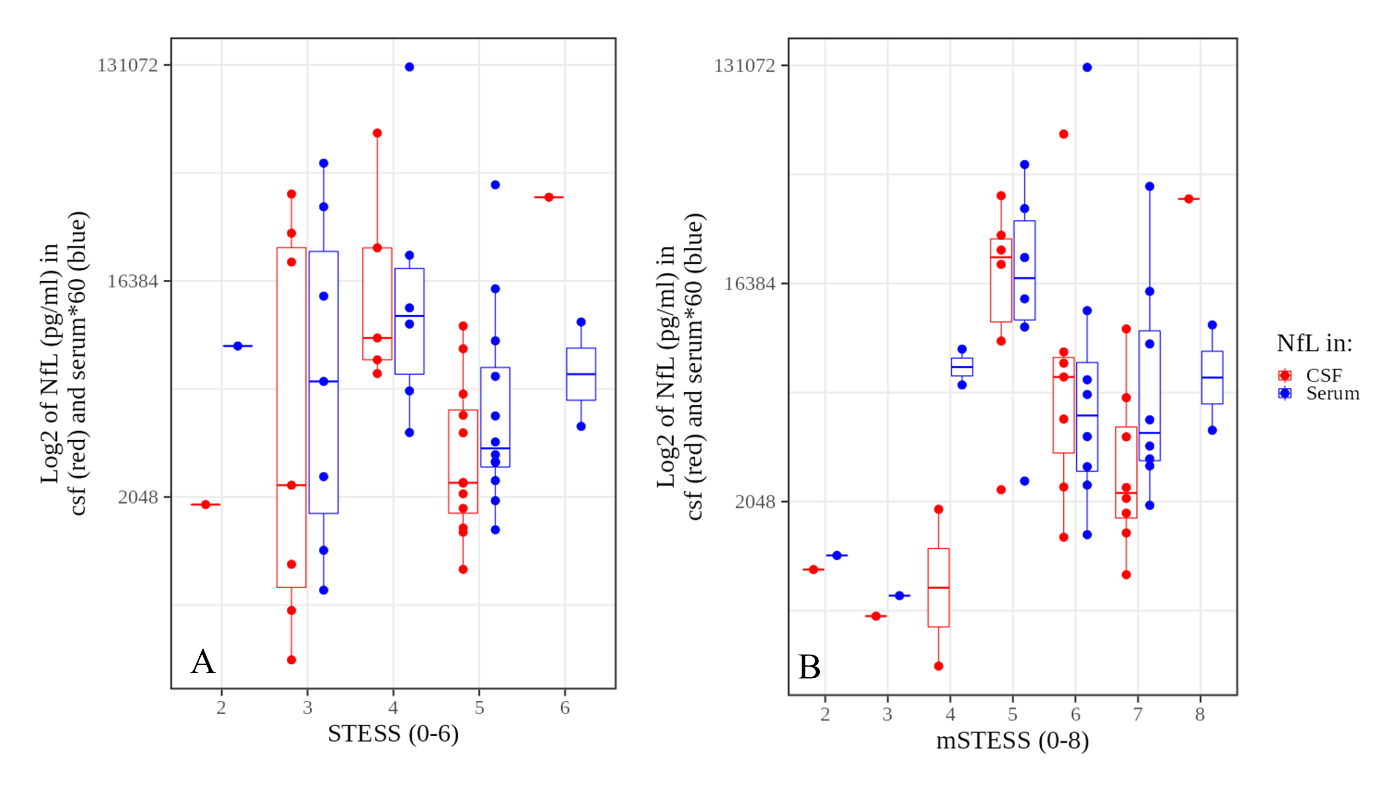

Supplement: Supplementary file 1 — Supplementary file1 (DOCX 491 kb) [file 415_2022_11547_MOESM1_ESM.docx]
